# Condensed-matter chemistry: from materials to living organisms

**DOI:** 10.1093/nsr/nwy128

**Published:** 2018-11-10

**Authors:** Ruren Xu, Kui Wang, Gang Chen, Wenfu Yan

**Affiliations:** 1 State Key Laboratory of Inorganic Synthesis and Preparative Chemistry, College of Chemistry, Jilin University, China; 2 State Key Laboratory of Natural and Biomimetic Drugs, Department of Chemical Biology, School of Pharmaceutical Sciences, Peking University, China; 3 Key Laboratory of Physics and Technology for Advanced Batteries (Ministry of Education), College of Physics, Jilin University, China

We have proposed, in a recent essay entitled ‘Towards a new discipline of condensed matter chemistry’ [1], a new research field to study the functionalities and chemical reactions of condensed matter [2] with multi-level structures, characterized by strong intermolecular forces and local organizational order. In this perspective, we suggest possible ways to study their properties and reactions in relevance to specific organizational characteristics of their surroundings in the condensed-matter states, using solid-state materials with special functions and living organisms with complex high-order structures, specifically solid-state superconductive materials, catalysts and biological condensed materials (BCMs) in the context of synthetic and biological chemistry, as illustrative examples. Furthermore, we explore how such condensed-matter-based studies may lead to deeper understanding and insights about fundamental chemistry problems important to materials science and life sciences.

## MATERIALS IN CRYSTALLINE STATES

Superconductivity is a fundamental property of certain materials [3]. Great efforts have been made for decades to attain superconductivity at a practically feasible temperature. The first superconductor with critical temperature (*T_c_*) over 77 K, yttrium barium copper oxide (YBCO, YBa_2_Cu_3_O_7−_*_x_* (*x* = 0 to 1)) has been studied extensively. The Cu oxidation state in this compound is determined by the oxygen stoichiometry. Its *T_c_* reaches the maximum (92 K) when *x* ≈ 0.15 and the structure is orthorhombic; and the superconductivity disappears at *x* ≈ 0.6, where the structure of YBCO changes from orthorhombic to tetragonal.

The composition of YBa_2_Cu_3_O_7_ can be written as (Y^3+^)(Ba^2+^)_2_(Cu^2+^)_2_(Cu^3+^)(O^2−^)_7_, with one-third of Cu in the +3 state. When the oxygen content is reduced from 7 to 6.5, all Cu will change to the +2 state and the material is a semiconductor.

The unit cell of YBa_2_Cu_3_O_7_ consists of three pseudo-cubic perovskite unit cells. All the corner sites of the unit cell are occupied by Cu, which has two different types of coordination—Cu(1) and Cu(2)—with respect to oxygen. And there are four possible crystallographic sites for oxygen: O(1), O(2), O(3) and O(4). The tripling of the perovskite unit cell leads to nine oxygen sites, whereas YBa_2_Cu_3_O_7_ has seven, and is therefore referred to as an oxygen-deficient perovskite structure.

The parent structure, YBa_2_Cu_3_O_7_, contains layers of Cu–O sheets and layers with Cu–O chains. The CuO_2_ layers and the Cu–O chains are known to play important roles for superconductivity. From *x* = 0 to *x* ≈ 0.2, *T_c_* remains around 90 K, whereas *T_c_* approaches a plateau at 60 K when *x* = 0.3–0.4, whereas, between the two ranges when *x* = 0.25, there are alternately ‘full’ and half oxygen chains. When *x* = 0.5 (i.e. YBa_2_Cu_3_O_6.5_), *T_c_* is found to be at 45 K with fully oxygenated Cu–O chains (as in YBa_2_Cu_3_O_7_) observed along the *b*-axis, alternating with fully vacant O(4) sites (as in YBa_2_Cu_3_O_6_).

It has been observed that the total oxygen stoichiometry decreases smoothly with the increasing sintering temperature. The oxygen atoms that are removed from the structure are exclusively located at the O(4) sites with (0,1/2,0). *In situ* neutron powder diffraction analysis has revealed those the positions O(5) at (1/2,0,0) are gradually filled as the sintering temperature increases; and, when the occupancies of the two sites become equal, the symmetry of the structure changes from orthorhombic to tetragonal. For *x* < 0.5, the symmetry is orthorhombic Pmmm, and sites O(4) and O(5) are not equivalent. When *x* = 0, sites O(4) are completely filled and sites O(5) are empty.

The transition temperature from the orthorhombic to the tetragonal phase depends on the oxygen partial pressure of the atmosphere of the experiment. It is ∼700°C in case of 1 atmosphere of oxygen partial pressure, and decreases with lowering oxygen partial pressures. The oxygen stoichiometry at the transition is always *x* ≅ 0.5, so that the orthorhombic phase exists over the range 0 ≤ *x* < 0.5, and the tetragonal phase over the range 0.5 < *x* ≤ 1.0.

Studies of the superconducting properties as a function of the oxygen content show that, for the orthorhombic form, *T_c_* decreases as the oxygen is removed from the structure, and it becomes zero as the crystallographic transition to the tetragonal phase is completed. Superconductivity has not been found in the tetragonal phase for any stoichiometry.

Study of the Hi-*T_c_* superconductor shows that *T_c_* depends on the preparation condition of the materials. To obtain superconductivity, one must develop a preparation and synthesis route to ensure that the product will have the required stoichiometry of oxygen in YBCO as described above.

## CATALYTIC MATERIALS WITH RICH CONDENSE MATTER CHEMISTRY

### Zeolite and porous catalysts

In the early 1950s, molecular sieves (zeolites) were first introduced to the catalytic industry for the refining of petroleum. Since then, considerable advances have been made in the development of new types of zeolites and porous materials from micro-, meso- to macroporus and MOF, COF materials. Many new catalytic processes were developed, including those for petroleum chemicals, fine chemicals and medicinal chemicals, among others. Parallel to these technological developments, rules and laws have been derived and discovered. We have found that the catalytic functions, such as activities and selectivity, are not only determined by channel structures (size, dimension and shape) and catalytic active sites of the Brönsted and Lewis solid acids, but also by their multi-level structural organization in solid state. For zeolites and porous materials, the defects, intergrowth, disorder and Al locations as well as the synthetic chemistry (e.g. hydrothermal crystallization and templating) including secondary synthesis and precise modification all make differences in terms of their catalytic functions. All these synthetic reactions take place in specific condensed-matter states such as aqueous, organic solvents and hydro-/solvo-thermal conditions, or during transitions across different states. Knowledge via mining-relevant databases could enable us to understand the relationships between catalytic functions, multi-level structures and synthesis routes and conditions of these materials, serving as the foundation for further studies. The big challenge to twenty-first-century catalysis science is to understand how to rationally design and synthesize the catalytic structures for needed activities and selectivity, representing the basis for the development of ‘condensed-matter engineering’ [4].

### Single-site catalyst

With the recent advances in catalysis chemistry and the catalytic industry, a large number of catalytic materials have emerged, aimed to improve catalysis efficiency as well as the scope of applicability. Two general directions exhibited effectiveness. One is to alter the condensed-matter state of the catalytic material, evolved from macroscopic solids, mesoscopic particles to single-site catalytic materials. In such material, catalysts (atom, cluster, molecule or functional group) are immobilized on nanosheets such as 2D materials [5] as well as graphene and its derivatives [6] in such ways as to maximize the reaction surface as well as the catalytic efficiency. The other is to take advantage of the specific properties of the 2D material such as defects, electronic and band structure [5,6] to provide synergistic supports to the single-site catalysts [5,7,8]. Research in such efforts has become an important direction of catalysis science [6,9].

### Photocatalyst

Studies of photocatalytic reactions and material have become more and more popular in recent years. Defects coupled with solar or other energy-intensive light have been utilized to form surface catalytic active sites via ‘defect engineering’, including (i) refining defect states in W_18_O_49_ by Mo doping allows N_2_ adsorption and photo-excited electrons to efficiently transfer from refining defect active-site into the anti-bonding π-orbitals of N_2_, which can better impel the N≡N bond dissociation and N_2_ reduction for running N_2_ activation towards solar-energy-driven nitrogen fixation [10]; (ii) photocatalytic conversion of N_2_ to NH_3_ with H_2_O at the surface oxygen vacancies in TiO_2_; and (iii) oxide defect engineering enables coupling solar energy into O_2_ activation. Single-site catalysis and photo-catalysis have contributed greatly to the conversion of small gaseous molecules such as CO_2_, CO, CH_4_ and H_2_ and lower olefins with high activity and selectivity. Extensive studies have been conducted regarding the catalytic activity and selectivity, the active-site and multi-level structures in solid state, and their precise synthesis and preparation, which has contributed to the development of condensed-matter engineering.

## LIVING ORGANISMS: BIOLOGICAL CONDENSED STATE

Biological macromolecules in the form of BCMs play pivotal roles in keeping organisms alive. Some act as the building blocks of cells and tissues, some incorporated in the biomembrane act as receptors, channels, transporters and signal transducers, and some others located in extra- or intracellular fluids.

Unlike the inorganic counterparts, a variety of high-order structures of BCMs were generated by the dynamic self-organization process. Differing from chemical self-assembly, it generates spatiotemporal order from the intrinsic disorder in a multiple molecular system and maintains the steady state by dissipating energy from nucleoside triphosphates (NTP). The dissipative structures thus generated make up different patterns, by which they display different functions [11]. The common features of biological condensed states might be summarized as the following aspects:

1. A variety of high-order structures of BCMs are assembled from available biomolecules for diverse functions, such as cell components, extracellular matrix (ECM) and secretions, among others. In most cases, the molecules are assembled and further organized by self-organization [11].
2. Most BCMs possess the feature of complex soft matters, characterized by their viscoelasticity, and take the form of sol/gel, liquid-like matter with multi-level structures built up from basic biomolecules [12]. The soft-matter properties are achieved mainly by non-covalent intermolecular interactions. In the supramolecular assemblies, such as cell membranes, the vulnerable conformation, flexibility, mobility and intermolecular interaction also contribute to the soft-matter properties. So much of the blood plasma is a more complex soft matter, and its rheology is partially attributed to its component proteins.
3. Most BCMs are located and act in aqueous media, and hence their structural conformation (such as protein folding) and reactivity depend on the surrounding solution; and, in reverse, BCMs as background may affect the reactions and subsequent events in the solution. The surrounding solutions are complicated by the crowding effect due to high concentrations of macromolecules in extra- or intracellular fluids and high density of proteins/lipids in the membrane. The crowding effect is further enhanced by the confinement effect due to compartmentalization of the intracellular space and the clustered extracellular matrix. Crowding coupled with confinement may have driving roles in self-organization, and may affect the conformation of macromolecules and influence diffusion and competition among different reactants and reaction specificities.
4. The biological interface represents probably the most important arena of the BCMs involved in chemical reactions and biological events, such as reactions at the cell–solid (like tooth and bone), cell–nanoparticle, cell–biofluid and cell–cell interfaces. Cells may attach to the large inorganic solid surface and initiate a sequence of events, such as proliferation, differentiation and apoptosis, during which some chemical reactions might be brought about. On the other hand, as the inorganic nanoparticles contact a cell, they might be engulfed by the cells or just deposit on the cell surface. They are both bioactive to induce further actions.

The features of BCM might be illustrated as follows.

Blood plasma might be considered as a suspension of blood cells in a concentrated solution of serum proteins. When blood passes through the jammed blood vessels, particularly capillaries, the rheological properties of blood play critical roles. The rheology of blood is mainly attributed to: high concentration of proteins displaying soft-matter properties, deformability of the erythrocytes and the viscoelasticity of the cell membrane. In turn, the deformability of erythrocytes is attributed to the self-regulated stretchable membrane skeleton, the crowding of hemoglobin molecules in the cell and the flexibility of the plasma membrane.

The mucus attached on the surface of the respiratory, digestive and reproductive tracts is a self-organized viscoelastic gel-mesh 3D network made of crosslinks among the linear homopolymers of the protein (mucin). When pathogens diffuse through the mucosa layer to attach to cell surfaces, they will recruit more pathogens to form colonies, and then build up their own protecting meshwork from the linear homopolymers of exopolysaccharides, leading to biofilm formation. The battle between mucosa and pathogens exemplifies the importance of the biological interface for chemical reactions and biological events.

Plasma membrane–cytoskeleton co-mposite is a cleverly designed architecture integrating the flexible bio-membrane and the rigid cytoskeleton to support the cell morphology and various functions. Biomembrane is a special family of condensed materials with fluid-like features. They are made of lipid bilayers and intercalated proteins. Weak intermolecular interaction and the mobility of molecules endow the membrane with flexibility and heterogeneity, which are required for different functions. Different from the fluidic membrane, cytoskeleton is a relatively rigid network comprising microfilaments, microtubule and intermediate filaments, assembled via self-organization. The contractility of microfilaments is important for cell division, cell movement and cell response to the environment. The generation and extend–retract process are driven by the energy released by hydrolysis of NTP (GTP for tubulin assembly). Although the process has been simulated *in vitro*, the self-organization is not yet demonstrated experimentally [13].

Cytoplasm of all cells is a viscous gel-like fluid containing proteins and other biomolecules in very high concentrations. Molecular crowding plus confinement contributes to the conformation of biomolecules by affecting intermolecular attraction–repulsion and the stability of different conformations. They also contribute to the rates and fate of reactions by affecting the diffusion rates, creating local concentrations and dictating some competitions among different molecules. It is foreseeable that the crowding effect might be a necessary condition for certain macromolecular interactions such as microfilament formation.

Biomineralization is a complex process by which different composites of insoluble metal salts and matrices deposit. For humans, of highest concern is calcification, in which calcium phosphate is produced and deposits in extracellular matrix (e.g. bone or enamel) or ectopically in certain tissues (calcification of plaque). It was known that the reaction between calcium and phosphate ions starts from the formation of ion clusters to amorphous calcium phosphate, which as a precursor transforms to the thermodynamically stable crystalline hydroxyapatite. Ossifications generally proceed at the bone–osteocytes interface and the calcium phosphates are deposited in the ECM, while the matrix is secreted and then organized by the cells in parallel with the mineral deposit. For a long time, bone-grafting substitutes created through chemical syntheses have been extensively studied to mimic the composition and structures of hard tissues. However, numerous problems need to be solved before such substitutes can reliably replace human bones. Metal phosphate deposition in specific tissues is a prevalent medical problem. So far, it is not clear how the gadolinium contrast agents get to selected tissues and deposit there; and what impact such deposits may have on the cells and tissues, such as gadolinium-induced nephrogenic systemic fibrosis and gadolinium brain deposit.

Condensed-matter chemistry studies (i) functionalities (physics and chemistry) of materials in condensed states, (ii) multi-level structures of such materials and (iii) the relationships among the functions and chemical reactions, the structures and synthesis (synthesis and/or preparation, assembly, self-assembly, self-organization). A new perspective should be taken to understand the chemistry problems, taking into full consideration the complex environments where the chemistry takes place rather than molecular-level chemistry. Such studies should provide useful guidance towards the engineering of new functional materials with designed functionalities, leading to the development of ‘condensed-matter engineering’. As a consequence of the development and maturation of new condensed-matter-centric chemistry, new sub-areas and new research directions will emerge in the closely related disciplines such as materials science, life sciences and architecture (synthesis) science.
